# Does the rate of orthodontic tooth movement change during pregnancy and lactation? A systematic review of the evidence from animal studies

**DOI:** 10.1186/s12903-020-01223-2

**Published:** 2020-08-27

**Authors:** Moaza Omar, Eleftherios G. Kaklamanos

**Affiliations:** 1 Dubai Health Authority, Dubai, United Arab Emirates; 2 Hamdan Bin Mohammed College of Dental Medicine (HBMCDM), Mohammed Bin Rashid University of Medicine and Health Sciences (MBRU), Building 34, Dubai Healthcare City, Dubai, United Arab Emirates

**Keywords:** Pregnancy, Lactation, Orthodontic treatment, Rate of tooth movement

## Abstract

**Background:**

The changes in bone homeostasis observed during pregnancy and lactation could result in alterations in the rate of orthodontic tooth movement, but research in human subjects presents significant ethical and practical limitations. Our aim was to compare the amount of orthodontic tooth movement between pregnant/lactating or not animals.

**Methods:**

We searched without restrictions 8 databases and performed hand searching until July 2019 (PubMed, Central, Cochrane Database of Systematic Reviews, SCOPUS, Web of Science, Arab World Research Source, ClinicalTrials.gov, ProQuest Dissertations and Theses Global). We searched for studies comparing quantitatively the amount of orthodontic tooth movement between pregnant/lactating or not animals. Following retrieval and selection of studies, the collection of related data was performed and the risk of bias was assessed using the SYRCLE’s Risk of Bias Tool. Exploratory synthesis was carried out using the random effects model.

**Results:**

Four studies were finally identified raising no specific concerns regarding bias. One study showed that lactation increased the rate of tooth movement by 50 % [*p* < 0.05]. Although an overall increase was noted in the pregnancy group as well, it did not reach statistical significance [3 studies, Weighted Mean Difference: 0.10; 95% Confidence Interval: − 0.04 - 0.24; *p* = 0.165].

**Conclusions:**

The metabolic changes occurring during pregnancy and lactation may have an impact on the rate of tooth movement in animals. Although these animal experimental results should be approached cautiously, it could be safe practice to consider the impact of these physiological changes in the clinical setting.

**Registration:**

PROSPERO (CRD42018118003).

## Background

During pregnancy females experience physiological changes associated with increases in oestrogen and progesterone levels, which lead to functional and tissue metabolism alterations critical to ensure a healthy gestation [1]. Regarding the skeletal system, the needs for maternal minerals increase in order to fulfil the mineralization of the developing foetal skeleton [2]. As a response, the calcium content of the maternal skeleton augments during the initial stages of pregnancy; later small reductions in bone mineral density might also be observed [3].

It is well recognised that oestrogen levels exert a critical role regarding bone mass preservation during gestation [4]. Oestrogen receptors have been observed in human cells [5] and several lines of evidence support that inhibition of bone remodelling by oestrogen is a result of osteoclastogenesis prevention from marrow precursors, as well as by induction of the Fas/FasL system that leads to osteoclast apoptosis [6, 7]. Oestrogen exerts a further inhibitory role on bone resorption through effects on the receptor activator of nuclear factor-Kappa B (RANK)/RANK ligand (RANKL)/osteoprotegerin (OPG) system and the production of some pro-resorptive cytokines (e.g. IL-1, IL-6, IL-7, TNF) [8–14]. However, oestrogen also affects directly the cells of the osteoblastic lineage contributing to bone preservation [15, 16].

Progesterone has also been shown to exert bone protective effects [17]. These results seem to be moderated directly via progesterone receptors in osteoblasts [18], as well as indirectly by acting as a ligand to the glucocorticoid receptor [17, 19]. Furthermore, progesterone may participate in the regulation of bone matrix, through its inhibitory action on metalloproteinases [20, 21].

Following pregnancy, lactation constitutes an important part of mammalian reproduction by ensuring the continuation of the supply of nutrients to the offspring [22]. The preparation of the female body begins already from pregnancy with increasing prolactin levels [23]. During lactation, prolactin that plays the principal role in stimulating the proliferation and differentiation of mammary cells [24], acts also as a key regulator of bone resorption by modulating sex hormone level [25, 26]. In general, lactation is characterized by a phase of oestrogen deficiency and attenuation of its bone protective effects [27]. Also, increases in osteoclasts are observed and overall bone remodelling alters in the direction of bone mass reduction [28]. In addition to oestrogen deficiency, other mechanisms including fluctuations in the levels of androgens and direct effects of prolactin on bone metabolism have been implicated with bone loss in women during lactation [29, 30]. At the same time, the requirements from the maternal system continue to be increased as the new-born gains minerals from the mother. If the dietary sources are insufficient, then a greater amount will be drawn from maternal skeletal sources, an event that could further affect negatively the maternal skeletal structure and lead to additional loss of bone mass [2].

As orthodontic tooth movement can be modulated by any condition that is implicated in the associated molecular pathways [31], the adaptive changes in bone homeostasis and the alterations in the balance between osteoclastic bone resorption and osteoblastic bone deposition observed during pregnancy and lactation could result in alterations in terms of the rate of tooth movement. However, to the best of our knowledge, this information has yet to be summarized in an evidence-based manner. As research in human subjects during these periods presents significant ethical and practical limitations, the use of animal models may provide a mean to improved understanding.

### Objective

The objective of the present review was to systematically investigate and appraise the quality of the most up to date available evidence regarding the differences in terms of the rate of orthodontic tooth movement between pregnant/lactating or not animals.

## Methods

### Protocol and registration

Initially a special protocol was developed (registration in PROSPERO: CRD42018118003) [32]. Regarding conduct and reporting we adhered to relevant methodological guidelines [33–35]. As the present study was a systematic review, ethical approval was not required.

### Eligibility criteria

The eligibility criteria were defined according the Participants, Intervention, Comparison, Outcomes and Study design domains (Table 1). We aimed to include prospective studies that compared quantitatively the amount of orthodontic tooth movement between pregnant/lactating or not animals of any kind [36]. We excluded the following types of studies: investigation on humans; studies involving animals subjected to additional clinical interventions such as tooth extraction, animals under medication, animals with pathological conditions or dietary deficiencies, like calcium deficiency that leads to additional decrease in bone density [37]. Also, we excluded ex vivo, in vitro, in silico studies; case studies; cross-over studies and studies without a separate control group; reviews (traditional reviews, systematic reviews and meta-analyses) and studies with less than 5 subjects per group analysed, based on relevant methodological suggestions [36].

**Table 1:** Eligibility criteria

Domain	Inclusion criteria	Exclusion criteria
**Participants**	Female animal subjects during pregnancy or lactation undergoing orthodontic tooth movement.	Male animal subjects Female animal subjects under medication, with pathological conditions or dietary deficiencies.
**Interventions**	All types of orthodontic interventions to induce movement of teeth.	Other kinds of orthodontic interventions, like growth modification, etc. Subjects undergoing any kind of orthodontic tooth movement in conjunction with other clinical interventions such as tooth extraction, etc.
**Comparisons**	Female animal subjects not pregnant or lactating undergoing orthodontic tooth movement.	
**Outcomes**	Quantitative data regarding the amount of orthodontic tooth movement measured by various ways [directly or from plaster models with callipers, feeler gauges, etc.; from histological cuts directly on the optical microscope or from digital photos; radiographs of any kind i.e. lateral cephalometric radiographs, Cone Beam CT, micro-CT, etc.].	Qualitative assessments regarding the amount of orthodontic tooth movement.
**Study design**	Experimental prospective controlled studies with a separate control group (according to the Scottish Intercollegiate Guidelines Network algorithm for classifying study design (available at http://www.sign.ac.uk/assets/study_design.pdf).	Human studies Case studies, cross-over studies, studies without a separate control group. In vitro, ex-vivo or in silico studies. Reviews, systematic reviews and meta-analyses. Less than 5 subjects per group analysed [36].

### Information sources and search strategy

Following the development of detailed search strategies, the two authors searched the whole content in 8 electronic databases until July 2019 (PubMed, Central, Cochrane Database of Systematic Reviews, SCOPUS, Web of Science, Arab World Research Source, ClinicalTrials.gov, ProQuest Dissertations and Theses Global) (Supplementary Table 1). The searches were conducted without placing restrictions on language and were supplemented by reviewing the bibliography in any relevant paper retrieved. Moreover, we had planned to contact the responsible author in the event we needed some clarifications on the content of a potentially eligible paper.

### Study selection, data collection and data items

The two investigators assessed the retrieved records for inclusion separately without being blinded about the identity of the authors and kept a record on all decisions. Kappa statistics were not computed following relevant recommendations [34]. Subsequently, data extraction was carried out by filling in special forms the following items: bibliographic data; information on study design; animal and orthodontic mechanics characteristics; tooth movement measurement methodology and results.

### Risk of bias in individual studies

The risk of bias was assessed by the authors using the SYRCLE’s risk of bias tool [38]. In all the processes described above any disagreements were resolved by discussion.

### Summary measures, synthesis of results, risk of bias across studies and additional analyses

Data on the amount of tooth movement are continuous; thus, they were expressed as Weighted Mean Difference (WMD) accompanied by the 95% Confidence Intervals (CI). Exploratory synthesis for the effect of pregnancy on the amount of tooth movement at the point of the longest follow-up was carried out using the random effects model [39, 40]. The overlap of the 95% CI was inspected graphically and the I^2^ statistic was calculated [34]. Analyses were performed with Comprehensive Meta-analysis software 3.3.070 (©2014 Biostat Inc., Tampa, Florida, USA).

Based on the research protocol, subgroup analyses as well as analyses for “small-study effects” and publication bias were planned, but were not performed finally due to the lack of an adequate amount of data [34]. Despite the lack of extensive information, the quality of evidence was assessed following Guyatt et al. [41] in order to adopt a structured and transparent approach in formulating an interpretation of the evidence.

## Results

### Study selection

**Fig. 1:**
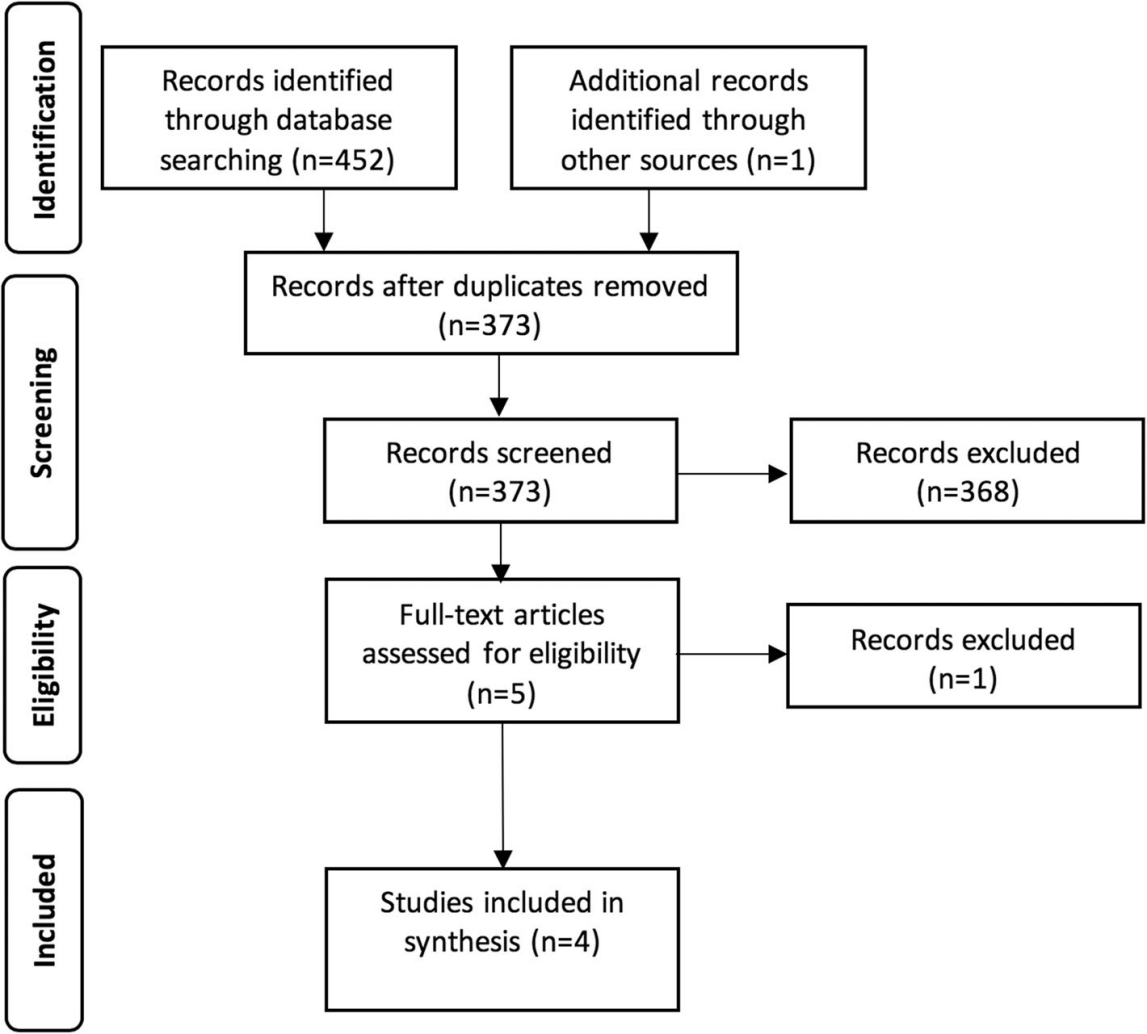
Flow diagram of the records through the reviewing process

Database search rendered 452 records and 1 record was located through hand searching. Later, we excluded 80 records as duplicates and 368 based on their title and abstract. After the exclusion of one more paper because it involved animals with dietary calcium deficiency [42], four papers were considered eligible (Fig. 1) [43–46].

### Study characteristics

The retrieved studies were published between 1991 and 2018 and investigated the influence of pregnancy [43–45] and lactation [46] on the amount of orthodontic tooth movement in rats and mice. Orthodontic tooth movement was induced by placing coil springs between maxillary incisors and molars or between incisors, as well as by using expansion arches on the molars, for periods of maximum 3 weeks. The rate of orthodontic tooth movement was assessed either clinically or radiographically from occlusal or lateral cephalometric radiographs, as well as micro-CT (Table 2). We tried to contact the corresponding authors of two studies for further information, but we are unable to get in touch with them [44, 46].

**Table 2:** Characteristics of the included studies

Study	Subjects & tooth movement model characteristics [number; age; weight]	Tooth movement measurements
**Ghajar et al.** [43] 2013 – Iran	48 Wistar rats [24 pregnant - 1^st^w; 24 control] 3 m; 250 ± 25 g; parity nm **Sample size calculation:** nm SS spring between Mx CIs [30 g] **Force application:** 2w	**Clinical measurements** Distance between the mesial corners of MxCIs^a^ **Method error assessment:** No
**Hellsing and Hammarström** [44] 1991 – Sweden	10 Sprague-Dawley rats [5 pregnant - beginning; 5 control] 3-5 m; 260 g on average; parity nm **Sample size calculation:** nm 0.011″ Australian wire between Mx FMs [15 g at 1 mm] **Force application:** 3w	**Measurements on occlusal radiographs** Transverse distance between the ends of the outer arms of the wire^b^ **Method error assessment:** Yes
**Kim and Lee** [45] 2000 - Korea	40 Sprague-Dawley rats [20 pregnant; 20 control] 10 w; 200-280 g; parity nm **Sample size calculation:** nm NiTi spring to between Mx CI and FM [40 g] **Force application:** 2w	**Measurements on lateral ceph. Radiographs** Mesial movement of the Mx FM^c^ **Method error assessment:** No
**Macari et al.** [46] 2018 – New Zealand & Brazil	12 C57BL/6 mice [6 lactating – d 9 postpartum; 6 control nulliparous] 15-17w **Sample size calculation:** nm NiTi spring between Mx CI and FM [35 g] **Force application:** 12d	**Measurements on micro-CT** Difference of the cemento-enamel junction distance of FM and SM between the control and experimental side^d^ **Method error assessment:** No

*CI* central incisor(s), *d* days, *FM* first molars, *Mx* Maxillary, *SM* Second molar, *SS* Stainless steel, *w* week(s), *nm* not mentioned

^a^Performed at 8 animals from each group at days 2, 7 and 14 of the experiment; ^b^Performed at 5 animals from each group at days 1, 4, 7, 11, 14, 18, and 21 of the experiment; ^c^Performed at 5 animals from each group at days 1, 3, 7 and 14 of the experiment; ^d^Performed at 6 animals from each group at day 12 of the experiment

### Risk of bias within studies

Table 3 presents the summary of findings regarding risk of bias assessment. For many domains there was insufficient information to permit judgements of low or high risk, but no important concerns were raised overall.

**Table 3:** Summary of risk of bias assessment

Study	Signalling questions									
	1	2	3	4	5	6	7	8	9	10
Ghajar et al., 2013 [43]	Unclear	Low	Unclear	Unclear	Low	Unclear	Low	Low	Low	Low
Hellsing and Hammarström, 1991 [44]	Unclear	Low	Unclear	Unclear	Low	Unclear	Low	Low	Low	Low
Kim and Lee, 2000 [45]	Unclear	Low	Unclear	Unclear	Low	Unclear	Low	Low	Low	Low
Macari et al., 2018 [46]	Unclear	Low	Unclear	Unclear	Low	Unclear	Low	Low	Low	Low

1: Was the allocation sequence adequately generated and applied?; 2: Were the groups similar at baseline or were they adjusted for confounders in the analysis?; 3: Was the allocation adequately concealed?; 4: Were the animals randomly housed during the experiment?; 5: Were the caregivers and investigators blinded to the intervention that each animal received?; 6: Were animals selected at random for outcome assessment?; 7: Was the outcome assessor blinded?; 8: Were incomplete outcome data adequately addressed?; 9: Are reports of the study free of selective outcome reporting?; 10: Was the study apparently free of other problems that could result in high risk of bias?

### Results of individual studies and synthesis of results

**Fig. 2:**
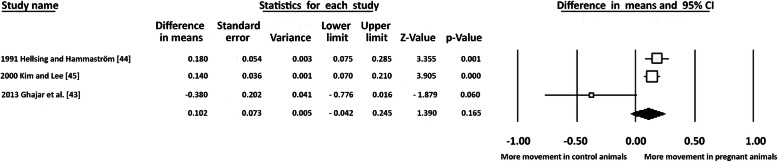
Forest plot for the exploratory data synthesis

Two studies showed more movement in the pregnant animals [44, 45] while no difference was observed in the third [43] (Fig. 2). Exploratory data synthesis showed an overall increase in tooth movement in the pregnancy group that did not reach statistical significance [WMD: 0.10; 95% CI: − 0.04 - 0.24; *p* = 0.165; I^2^ = 72%]. Regarding lactation, Macari et al. [46] reported a significantly greater amount of tooth movement in lactating animals compared to the control group by 50% [*p* < 0.05].

### Additional analyses and risk of bias across studies

It was not possible to conduct analyses for “small-study effects” and publication bias, nor for subgroup analyses. Regarding the effect of pregnancy and lactation on the amount of orthodontic tooth movement the quality of available evidence was considered as moderate (Supplementary Table 2).

## Discussion

### Summary of available evidence

The alterations in bone homeostasis occurring during pregnancy and lactation could possibly have an effect on the amount of orthodontic tooth movement. Based on the animal studies retrieved, lactation increased the amount of tooth movement. Exploratory synthesis showed an overall increase in the pregnancy group as well. However, this tendency did not reach statistical significance. Although these animal experiment results should be approached with some caution until more information becomes available, the clinician should not ignore the possibility that orthodontic patients during pregnancy or breastfeeding may exhibit changes in physiological bone remodelling, as well as the possible implications for clinical practice. Especially patients in lactation, might present increased needs for anchorage preparation during space closure. Furthermore, appointments might need to be more frequent in order to check and control the progress of treatment.

Quantitative synthesis of the information on pregnant and control animals revealed a tendency for increase in the rate of tooth movement. On the histological level, Hellsing and Hammarström [44] did not show a significant difference in the number of osteoclasts. Ghajar et al. [43] observed that the number of osteoclasts was significantly reduced in the pregnant rats, but on the clinical level the difference was not significant. The fact that paraffin histological analyses can only be performed in two dimensions might account for these differences in findings. Regarding osteoblasts, higher percentages have been observed in pregnant animals [47]. Kim and Lee [45] measured alkaline phosphatase and tartrate-resistant acid phosphatase activities in extracts of paradental alveolar bone, as a way to assess bone metabolism. Their results showed high activity in the pregnant group only at the early stages of the experiment. This information could suggest that, in the context of rat pregnancy that lasts 21–23 days [48] tooth movement could be promoted during pregnancy because the action of resorption is faster than deposition.

During pregnancy, the physiological maternal adaptations in the osseous metabolism result from the involvement of various regulators [49]. Oestrogens are known down-regulators of bone resorption and act to maintain bone mass [50]. In the context of orthodontic treatment, the administration of oestrogen reduced the rate of tooth movement in osteoporotic rats [51]. Progesterone also has been reported to lead to the same results directly through action on the osteoblasts, or indirectly by influencing the glucocorticoid receptors or the metalloproteinases [17] and has been linked with reduction in the rate of tooth movement [52]. On the contrary prolactin, which is present with increased levels during pregnancy, exhibits pro-resorptive action leading to reductions in bone mass [28]. A multitude of other hormones and biological factors have been implicated in the regulation of the processes associated with bone remodelling during pregnancy as well [49, 53, 54], which could potentially modify the rate of clinical movement under the influence of orthodontic forces.

Apart from the overall regulation of bone remodelling, local alterations in the periodontal tissues could account for the observed clinical changes. As periodontal ligament cells exhibit oestrogen receptors, the hormonal changes taking place in pregnancy might lead to water retention [55]. Thus, the periodontal ligament might become easily compressible in pregnant individuals when a mechanical force is applied. It is also expected that slight extrusion of the teeth will happen simultaneously which will facilitate the greater amount of tipping movement [44].

According to Macari et al. [46], lactation resulted in a significantly increased rate of tooth movement compared to the non-lactating group. Lactating animals exhibited elevated rates of bone turnover resulting in bone loss in the maxilla, femur and vertebra. These changes are consistent with those reported previously in long bones and the mandible of lactating calcium deficient mice [26, 29, 56–58] and can be associated with the bone mass reducing effect of prolactin [28]. On the contrary Shoji et al. [37] observed no effect of lactation on the density of the alveolar bone when calcium content of the diet is normal, while other researchers observed even increases in the height of alveolar bone [59]. Such discrepancies could be a result of the different methodologies employed.

Macari et al. [46] also observed that the osteopenic phenotype was associated with an increased expression of the RANK/RANKL/OPG signalling pathway in the alveolar bone. These findings were consistent with previous findings of increased expression of these factors in the calvaria of lactating mice [60] as well as prolactin treated osteoblast-like cells [61]. Increased bone turnover could also be attributed to the prolactin induced differentiation of osteoclasts [60]. Therefore, lactation associated alterations in the alveolar bone led to reductions in bone mineral density and to diminished trabecular bone architecture.

### Strengths and limitations

For this review we followed well-established guidelines in an attempt to reduce methodological bias and we focused our unrestricted and comprehensive searches on controlled trials. We also performed an exploratory quantitative synthesis that albeit indicative until additional research becomes available, it is more transparent and potentially more valid than alternative summaries [62]. It has been suggested that if meaningful, even data from two studies can be combined [34, 63].

Furthermore, it has to be acknowledged that the data retrieved in the present systematic review relate mostly to rodents and cannot be directly extrapolated to humans. Investigations based on rats and mice have given important physiological information. However, significant differences between rodents and humans exist, not only in terms of bone physiology, but also of pregnancy/lactation endocrinology [23, 64]. Also, one should not forget that the biomechanical conditions were various and not analogous to clinical scenarios in humans [65]. Finally, as power sample calculations were not included in the methodology, the precision of the retrieved results could be potentially questioned. Consequently, it cannot be determined with certainty what would be the effect in everyday clinical practice. However, analogous human studies present significant ethical and practical limitations.

### Recommendations for future research

Since, the number of adult female patients seeking orthodontic treatment appears to be on the rise, further well- designed experimental studies on the effects of pregnancy and lactation on orthodontic tooth movement would be useful for the clinician. It is highly desirable that study designs become standardized [66] and possible sources of risk of bias receive the appropriate attention [38]. Moreover, study designs should come closer to everyday clinical scenarios.

## Conclusions

The metabolic changes occurring during pregnancy and lactation in animals may have an impact on the rate of tooth movement. Although these animal experiment results should be approached cautiously, it could be safe practice to consider the possible impact of these physiological changes in the clinical setting.

## Supplementary information

**Additional file 1: Table S1.** Strategy for database search (up to July 18th 2019). **Table S2.** Quality of available evidence.

## Data Availability

All data and materials are available upon request.

## Abbreviations

CI: Confidence Interval
CT: Computed Tomography
IL: Interleukin
OPG: Osteoprotegerin
PICOS: Participants, Interventions, Comparisons, Study design
PROSPERO: International prospective register of systematic reviews
RANK: Receptor activator of nuclear factor-Kappa B
RANKL: Receptor activator of nuclear factor-Kappa B ligand
SYRCLE: SYstematic Review Center for Laboratory animal Experimentation
TNF: Tumor Necrosis Factor
WMD: Weighted Mean Difference
