# Supplementary material for: Does the rate of orthodontic tooth movement change during pregnancy and lactation? A systematic review of the evidence from animal studies
Source: BMC Oral Health. 2020 Aug 27;20:237. doi: 10.1186/s12903-020-01223-2 (PMC7450973; doi:10.1186/s12903-020-01223-2)
Supplement: Supplementary file 1 — Additional file 1: Table S1. Strategy for database search (up to July 18th 2019). Table S2. Quality of available evidence. [file 12903_2020_1223_MOESM1_ESM.docx]

**Supplementary Table 1.** Strategy for database search (up to July 18^th^ 2019).

| **Database** | **Search strategy** | **Hits** |
| --- | --- | --- |
| **PubMed** | (pregnancy OR gestation OR germination OR fertilization OR gravidity OR impregnation OR anticipating OR expecting OR fecundity OR parous OR parturient OR litter OR conception OR breeding OR begetting OR procreation OR reproduction OR parturiency OR maternity OR motherhood OR incubation OR birth OR nascency OR lactation OR suckling OR “breast feeding” OR “breast milk”) AND ("tooth movement" OR "orthodontic movement" OR "orthodontic anchorage") | **66** |
| **Cochrane Central Register of Controlled Trials**  via Ovid | ((pregnancy or gestation or germination or fertilization or gravidity or impregnation or anticipating or expecting or fecundity or parous or parturient or litter or conception or breeding or begetting or procreation or reproduction or parturiency or maternity or motherhood or incubation or birth or nascency or lactation or suckling or breast feeding or breast milk) and (tooth movement or orthodontic movement or orthodontic anchorage)).ti. or ((pregnancy or gestation or germination or fertilization or gravidity or impregnation or anticipating or expecting or fecundity or parous or parturient or litter or conception or breeding or begetting or procreation or reproduction or parturiency or maternity or motherhood or incubation or birth or nascency or lactation or suckling or breast feeding or breast milk) and (tooth movement or orthodontic movement or orthodontic anchorage)).ab. | **11** |
| **Cochrane Database of Systematic Reviews**  via Ovid | ((pregnancy or gestation or germination or fertilization or gravidity or impregnation or anticipating or expecting or fecundity or parous or parturient or litter or conception or breeding or begetting or procreation or reproduction or parturiency or maternity or motherhood or incubation or birth or nascency or lactation or suckling or breast feeding or breast milk) and (tooth movement or orthodontic movement or orthodontic anchorage)).ti. or ((pregnancy or gestation or germination or fertilization or gravidity or impregnation or anticipating or expecting or fecundity or parous or parturient or litter or conception or breeding or begetting or procreation or reproduction or parturiency or maternity or motherhood or incubation or birth or nascency or lactation or suckling or breast feeding or breast milk) and (tooth movement or orthodontic movement or orthodontic anchorage)).ab. | **0** |
| **Scopus** | TITLE-ABS-KEY((pregnancy OR gestation OR germination OR fertilization OR gravidity OR impregnation OR anticipating OR expecting OR fecundity OR parous OR parturient OR litter OR conception OR breeding OR begetting OR procreation OR reproduction OR parturiency OR maternity OR motherhood OR incubation OR birth OR nascency OR lactation OR suckling OR "breast feeding" OR "breast milk") AND ("tooth movement" OR "orthodontic movement" OR "orthodontic anchorage")) | **95** |
| **Web of Science™** | TOPIC: ((pregnancy OR gestation OR germination OR fertilization OR gravidity OR impregnation OR anticipating OR expecting OR fecundity OR parous OR parturient OR litter OR conception OR breeding OR begetting OR procreation OR reproduction OR parturiency OR maternity OR motherhood OR incubation OR birth OR nascency OR lactation OR suckling OR "breast feeding" OR "breast milk") AND ("tooth movement" OR "orthodontic movement" OR "orthodontic anchorage")); Timespan: All years. Databases: WOS, KJD, RSCI, SCIELO, ZOOREC; Search language=Auto | **239** |
| **Arab World Research Source** | TI tooth movement AND AB tooth movement | **3** |
| **ClinicalTrials.gov** | (orthodontic OR orthodontics) AND (tooth movement) | **37** |
| **ProQuest Dissertations and Theses Global** | ti((pregnancy OR gestation OR germination OR fertilization OR gravidity OR impregnation OR anticipating OR expecting OR fecundity OR parous OR parturient OR litter OR conception OR breeding OR begetting OR procreation OR reproduction OR parturiency OR maternity OR motherhood OR incubation OR birth OR nascency OR lactation OR suckling OR "breast feeding" OR "breast milk") AND ("tooth movement" OR "orthodontic movement" OR "orthodontic anchorage")) AND ab((pregnancy OR gestation OR germination OR fertilization OR gravidity OR impregnation OR anticipating OR expecting OR fecundity OR parous OR parturient OR litter OR conception OR breeding OR begetting OR procreation OR reproduction OR parturiency OR maternity OR motherhood OR incubation OR birth OR nascency OR lactation OR suckling OR "breast feeding" OR "breast milk") AND ("tooth movement" OR "orthodontic movement" OR "orthodontic anchorage")) | **1** |

**Supplementary Table 2.** Quality of available evidence.

| - **Quality assessment** | | | | | | - **Effect** | - **Quality** |
| --- | --- | --- | --- | --- | --- | --- | --- |
| - **Studies** | - **Risk of bias** | - **Inconsistency** | - **Indirectness** | - **Imprecision** | - **Other** |  |  |
| **Amount of orthodontic tooth movement in pregnant animals** | | | | | | | |
| - 3 | - Not serious | - Serious^1^ | - Not serious | - Not serious | - None | - No difference between groups | - ⨁⨁⨁◯ **MODERATE** |
| - **Amount of orthodontic tooth movement in lactating animals** | | | | | | | |
| - 1 | - Not serious | - Not serious | - Not serious | - Serious^2^ | - None | - Increase in the EG | - ⨁⨁⨁◯ **MODERATE** |

- EG: Experimental group
- ^1^Substantial heterogeneity and conflicting results were noted; ^2^The number of animals analysed was limited
